# Metabolomic and inflammatory mediator based biomarker profiling as a potential novel method to aid pediatric appendicitis identification

**DOI:** 10.1371/journal.pone.0193563

**Published:** 2018-03-12

**Authors:** Nusrat S. Shommu, Craig N. Jenne, Jaime Blackwood, Ari R. Joffe, Dori-Ann Martin, Graham C. Thompson, Hans J. Vogel

**Affiliations:** 1 Bio-NMR Center, Department of Biological Sciences, University of Calgary, Calgary, AB, Canada; 2 Calvin, Phoebe and Joan Snyder Institute for Chronic Diseases, University of Calgary, Calgary, AB, Canada; 3 Department of Pediatrics, University of Calgary, Calgary, AB, Canada; 4 Division of Pediatric Critical Care Medicine, Department of Pediatrics, University of Alberta, Edmonton, AB, Canada; 5 Department of Pediatrics and Emergency Medicine, University of Calgary, Calgary, AB, Canada; Oswaldo Cruz Foundation, BRAZIL

## Abstract

Various limitations hinder the timely and accurate diagnosis of appendicitis in pediatric patients. The present study aims to investigate the potential of metabolomics and cytokine profiling for improving the diagnosis of pediatric appendicitis. Serum and plasma samples were collected from pediatric patients for metabolic and inflammatory mediator analyses respectively. Targeted metabolic profiling was performed using Proton Nuclear Magnetic Resonance Spectroscopy and Flow Injection Analysis Mass Spectrometry/Mass Spectrometry and targeted cytokine/chemokine profiling was completed using a multiplex platform to compare children with and without appendicitis. Twenty-three children with appendicitis and 35 control children without appendicitis from the Alberta Sepsis Network pediatric cohorts were included. Metabolomic profiling revealed clear separation between the two groups with very good sensitivity (80%), specificity (97%), and AUROC (0.93 ± 0.05) values. Inflammatory mediator analysis also distinguished the two groups with high sensitivity (82%), specificity (100%), and AUROC (0.97 ± 0.02) values. A biopattern comprised of 9 metabolites and 7 inflammatory compounds was detected to be significant for the separation between appendicitis and control groups. Integration of these 16 significant compounds resulted in a combined metabolic and cytokine profile that also demonstrated strong separation between the two groups with 81% sensitivity, 100% specificity and AUROC value of 0.96 ± 0.03. The study demonstrated that metabolomics and cytokine mediator profiling is capable of distinguishing children with appendicitis from those without. These results suggest a potential new approach for improving the identification of appendicitis in children.

## Introduction

Appendicitis is a common pediatric disease reaching peak incidence during the second decade of life among children [1–4]; it is characterized by increased bacterial growth inside the appendix that causes inflammation [5–8]. With early diagnosis and proper management, patient outcomes are most often excellent; however, delayed diagnosis can lead to perforation that may result in severe complications, including peritonitis, sepsis, bowel obstruction, abscess formation and fertility problems. Perforated appendicitis has been shown to increase the mortality rate by several fold [9–12]. However, clinicians must balance efforts to reduce delayed/missed diagnoses with over-diagnosis resulting in needless interventions (medical/surgical); recent studies show that current pediatric negative appendectomy rate is approximately 6% in Canada and USA [4,13].

Multifaceted barriers have the potential to hinder timely and accurate diagnosis of pediatric appendicitis. In many cases children do not present with the characteristic clinical features of appendicitis, which may challenge accurate diagnosis [1]. In addition, while older children can describe pain and provide a history of their illness, infants, toddlers and pre-school children may not have the capacity to do so in an accurate manner, which can result in misleading clinical history [1,2,14,15]. As a matter of fact, children under 5 have the highest (17%) missed diagnosis rate [16]. Imaging studies are often included in the diagnostic process for pediatric appendicitis, however these may be limited by availability, accuracy or exposure to radiation [17,18].

The present study focused on developing an integrated metabolomics and inflammatory mediator based approach and investigating its potential for the diagnosis of appendicitis in children. Metabolites are the intermediate and end products of cellular metabolic processes within an organism under any given physiological condition; hence metabolomics can provide a unique insight by analyzing low molecular weight compounds in biological systems [19]. Advancement of analytical techniques such as nuclear magnetic resonance (NMR) and mass spectrometry (MS) has enabled the quantitative identification of a wide range of metabolites using a very small volume of biofluid sample; it is also possible to compare the metabolic responses under different physiological conditions, providing better insight into any disease mechanism and identifing potential biomarkers for diagnosis [20]. Metabolic profiling has been successfully applied to a wide range of studies including infectious disease research [21,22]. Similar to metabolomics, multiplexed cytokine/chemokine analysis allows for the quantitative measurement and comparison of a broad range of inflammatory mediators that aids in creating a better understanding of the immune response under any biological condition [23,24]. In previous studies by our group, a combination of metabolomics and inflammatory mediator profiling has been shown to be useful for the diagnosis and prognosis of sepsis in the ICU in both adult and pediatric patient groups [25,26]. In the present pilot study, we have integrated quantitative metabolic and inflammatory mediator data as a potential novel approach to aid in the identification of pediatric appendicitis.

In an effort to decrease negative appendectomy rates (NAR), reduce misdiagnoses of alternate pathologies and eliminate the risks of unnecessary anesthesia and surgical intervention, the vast majority of children in North America undergo both laboratory (standard markers of infection/inflammation–WBC, neutrophils, CRP) and imaging investigations (ultrasound, CT, MRI). We feel that the long-term benefit of establishing a pediatric appendicitis biomarker profile is the potential for future collaboration with industrial partners to help create a clinical tool that will result in a decreased reliance on painful (US probing), costly, radiating (CT), imaging studies that are often not available in peripheral/rural locations.

## Materials and methods

### Patient enrollment

Approval for this study was obtained from the Health Research Ethics Board of the University of Alberta and the Conjoint Health Research Ethics Board of the University of Calgary. Written consent forms, approved by both the ethics boards was signed by the parents/guardians of the enrolled children. We performed a case-control analysis of a subset of pediatric patients aged 0–17 years who were prospectively enrolled in the Alberta Sepsis Network (ASN) cohort, which was a parent study aimed at elucidating the underlying metabolic and inflammatory processes occurring in children receiving care in the Emergency Department (ED) or the Pediatric Intensive Care Unit (PICU) for an infectious illness [26]. The *appendicitis cohort* included children from the ASN study with a hospital or ED discharge diagnosis of appendicitis, which was confirmed by pathology examination, both perforated and simple. Perforated appendicitis was determined when there was evidence of inflammation of the appendix along with any presence of perforation whereas simple appendicitis was determined when there is evidence of inflammation of the appendix without presence of perforation according to the pathology report. The *control cohort* included children from the ASN study presenting in the Alberta Children’s Hospital ED who did not a) show any clinical signs of infectious disease, b) undergo any procedure related to infection, c) report fever in the preceding 2 weeks, d) have any medical history that may influence the immune system, and e) take any immunosuppressive drug in the preceding 2 weeks. Blood samples were collected from the children before they received any antibiotics or any sedative medication; the detailed procedure for sample collection is described in previous ASN studies for pediatric sepsis [26]. In brief, blood was collected into two separate tubes for obtaining serum for metabolic analysis and plasma for cytokine analysis. After centrifugation at cold temperature both serum and plasma were immediately frozen at -70°C in 4ml cryovials and distributed for downstream analysis.

### Targeted metabolic profiling

Proton Nuclear Magnetic Resonance (^1^H NMR) Spectroscopy and Flow Injection Analysis Mass Spectrometry/Mass Spectrometry (FIA MS/MS) approaches were applied for targeted metabolic profiling. ^1^H NMR spectra were obtained using a 600 MHz Bruker Ultrashield Plus NMR spectrometer (Bruker BioSpin Ltd., Milton, ON, Canada) following procedures described previously [25,26]. Briefly, serum samples were filtered to remove large molecules using 3kDa NanoSep microcentrifuge filters (Pall, Inc., East Hills, NY, USA) followed by addition of phosphate buffer, sodium azide and D_2_O to the filtrate. ^1^H NMR spectra were obtained using a standard Bruker 1D spectroscopy pulse program ‘noesypr1d’ [27,28]. Each sample spectrum was individually processed and profiled using Chenomx NMR Suite 7.5 software (Chenomx Inc., Edmonton, Canada); metabolites were detected using the Chenomx library [28]. FIA MS/MS was performed commercially at Chenomx Inc., Edmonton, Canada using the AbsoluteIDQ^TM^ p150 kit (Biocrates Life Sciences AG, Innsbruck, Austria). Sample preparation was done following the manufacturers protocol that has been previously described [29]. In brief, 10μL serum was centrifuged and inserted into a filter on a 96-well sandwich plate containing stable isotope-labeled internal standards. After sequential derivatization, centrifugation and dilution the final extracts were analyzed by FIA-MS/MS, and targeted metabolites were quantified using internal standards.

### Inflammatory mediator profiling

Two human cytokine and chemokine assay kits (Bio-Plex Pro Human Cytokine 21-plex Assay and Bio-Plex Pro Human Cytokine 27-plex Assay) were used to analyze the inflammatory mediators in human plasma samples. The kits were obtained from Bio-Rad Laboratories, Inc. (Hercules, CA, USA) and the plates were read on a Luminex 200 apparatus (Applied Cytometry Systems, Sheffield, UK). Samples were assayed following the manufacturer guidelines; acquisition and analysis of the samples were done using Bio-Plex Manager 6.0. (Bio-Rad Laboratories, Inc.). The data was considered as a missing value if the coefficient of variance between two replicates was greater than 20%.

### Multivariate statistical analysis

SIMCA-P+ software (v12.0.1; Umetrics, Umeå, Sweden) was used for multivariate statistical analysis [20,30–34]. Metabolites detected by the ^1^H NMR and FIA MS/MS platforms were combined and the metabolites and inflammatory mediators that had >50% missing values were excluded from the statistical analysis. The average concentrations were taken for the common metabolites detected from both ^1^H NMR and FIA MS/MS approaches. Data preprocessing included median fold change normalization, logarithmic transformation, centering, and unit variance scaling for both the metabolite and protein mediator data sets [34]. An initial impression of the datasets was obtained through an unsupervised principal component analysis (PCA), which also showed the outliers situated outside the 95% confidence interval that could substantially affect the supervised models [30]. Supervised orthogonal partial least squares discriminant analysis (OPLS-DA) models were developed excluding the outliers to identify separation between the appendicitis and control groups [30,31]. A sevenfold cross-validation method was used to calculate the variation (R^2^Y) and predictive ability (Q^2^) [30,35]. Only those metabolites and protein mediators with variable importance to projection (VIP) > 1 were considered in building the OPLS-DA models [30]. P-values and the receiver operating characteristic curve (ROC) (Metz ROC Software; University of Chicago, Chicago, IL USA) were calculated in order to validate the OPLS-DA models [36,37]; sensitivity, and specificity were calculated from sample class prediction during sevenfold cross-validation (YpredCV). In addition, important metabolites and inflammatory mediators having significant concentration differences (p <0.05) between the diseased and control groups were identified from the OPLS-DA regression coefficients [30]. Subsequently, these significant metabolites and inflammatory mediators were combined to develop an integrated model. Data processing steps for the combined dataset were similar to that of the individual metabolic and cytokine datasets, which included median fold change normalization, logarithmic transformation, centering, and unit variance scaling. Both unsupervised (PCA-X) and supervised (OPLS-DA) models, as described above, were built for the combined dataset.

## Results

### Sample description

A total of 23 appendicitis and 35 control patients from the ASN pediatric cohorts were included in the metabolic and inflammatory protein mediator analyses. The demographic and clinical characteristics of the included children are demonstrated in Table 1. A total of 144 metabolites from ^1^H NMR spectroscopy and FIA MS/MS techniques and 56 inflammatory compounds from the Luminex platform could be reliably detected and analyzed for each patient. The list of all the detected compounds is provided in the supporting S1 Table.

**Table 1:** Demographic and clinical description of the appendicitis and control patient cohorts.

	Appendicitis Cohort (n = 23)	Control Cohort (n = 35)
**Age, average (SD)**	9.7 (4.6)	8.5 (4.3)
**Sex**		
Male, n (%)	13 (57%)	24 (69%)
Female, n (%)	10 (43%)	11 (31%)
**CTAS, median (IQR)**	3 (1)	3 (1)
**WBC, median (IQR)**	14.4 (10.1)	N/A
**Perforation of the appendix, n (%)**	11 (48%)	N/A
**Reason for Sedation**		
Fracture Reduction, n (%)	N/A	27 (77%)
Complex Laceration Repair, n (%)	N/A	3 (9%)
Other, n (%)	N/A	5 (14%)

### Metabolic profiling

**Fig 1:**
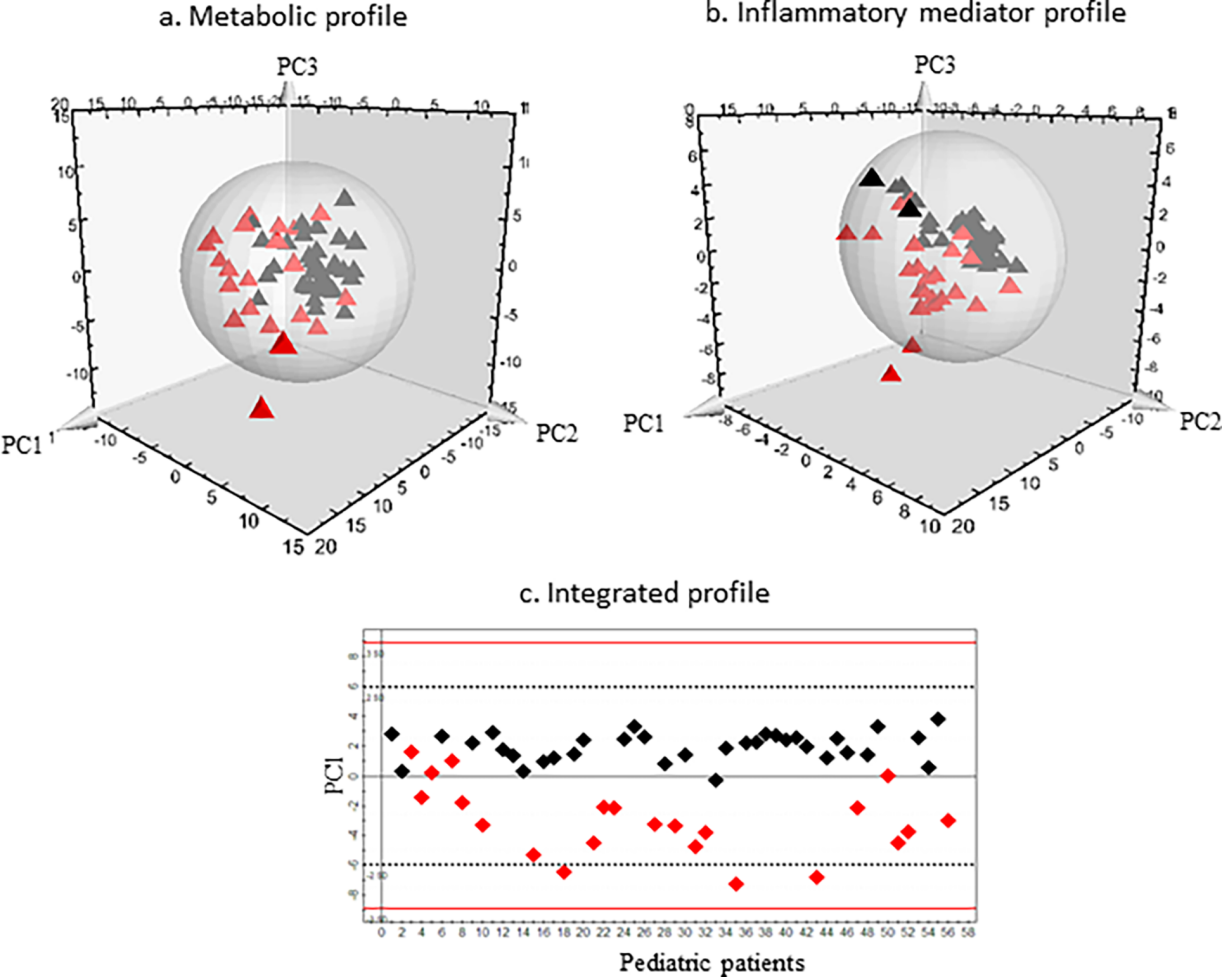
Three-dimensional PCA-X score scatter plots of pediatric patients with and without appendicitis using **(a)** metabolic profile and **(b)** inflammatory mediator profile and (**c**) integrated metabolic and inflammatory mediator profile, based on three principle components- PC1, PC2 and PC3. Red tetrahedrons represent the pediatric appendicitis patients and black tetrahedrons represent the pediatric control patients.

**Fig 2:**
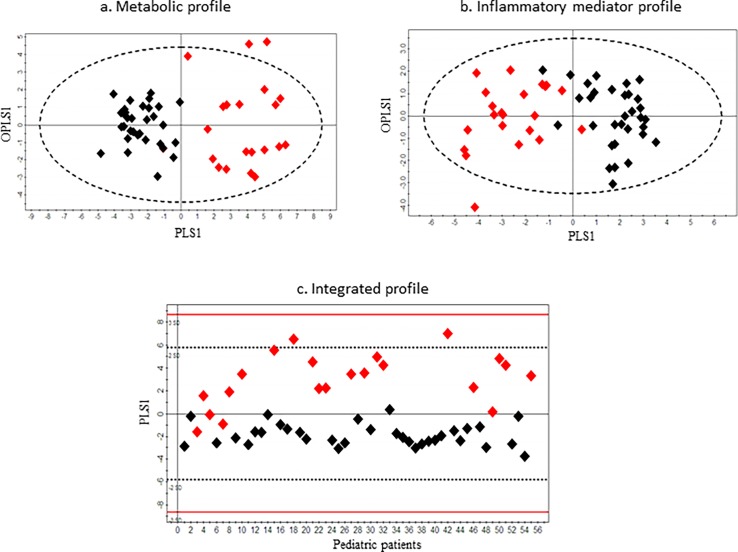
OPLS-DA score scatter plots distinguishing pediatric appendicitis patients from pediatric control patients using **(a)** metabolic profile (R^2^Y = 0.74, Q^2^ = 0.54), **(b)** inflammatory mediator profile (R^2^Y = 0.75, Q^2^ = 0.63), and (c) integrated metabolic and inflammatory mediator profile (R^2^Y = 0.69, Q^2^ = 0.67). Red diamonds represent the pediatric appendicitis patients and black diamonds represent the pediatric control patients.

The unsupervised PCA model for the metabolite dataset was built based on three principal components- PC1, PC2 and PC3, contributing to 17.9%, 12.02% and 8.9% percentages of the variation respectively (Fig 1A). Two outliers were identified that belonged to the appendicitis patient group and were excluded from further downstream analysis. The common metabolites that were detected from both ^1^H NMR and FIA MS/MS platforms demonstrated similar trends between the diseased and control groups. A supervised OPLS-DA model was built to illustrate the metabolic differences between appendicitis and control patient classes. The score scatterplot demonstrates substantial separation of the two groups with strong variance (cumulative R^2^Y = 0.74) and predictivity (cumulative Q^2^ = 0.54) (Fig 2A). The strength of the model was further supported by very good sensitivity (80%), specificity (97%), and AUROC (0.93 ± 0.05) values (Table 2).

**Table 2:** Summary statistics from OPLS-DA models differentiating pediatric patients with and without appendicitis.

Analysis	R^2^Y Q^2^	P-value	Sensitivity	Specificity	AUROC ± SD
Metabolomics	0.74 0.54	5.1E-08	0.80	0.97	0.93 ± 0.05
Protein mediators	0.75 0.63	3.8E-10	0.82	1.0	0.97 ± 0.02
Integrated profile	0.69 0.67	2.98E-13	0.81	1.0	0.96 ± 0.03

Abbreviations: AUROC- area under the receiver operating characteristic curve, SD-standard deviation

### Inflammatory mediator profiling

Similar to the metabolic dataset, three principal components (PC1, PC2 and PC3) were calculated to build the unsupervised PCA model for the inflammatory mediator dataset, which contributed 20.8%, 14.8% and 10.8% percentages of the variation respectively (Fig 1B). Three outliers were identified, of which one belonged to the appendicitis patient group and two belonged to the control patient group. As recommended by the Simca manual these outliers were excluded from the following supervised OPLS-DA analysis, which demonstrated the differences in protein mediator profiles between appendicitis and control patient classes. The model showed considerable separation of the two groups with high variance (cumulative R^2^Y = 0.75) and predictivity (cumulative Q^2^ = 0.63) (Fig 2B) along with remarkable sensitivity (82%), specificity (100%), and AUROC (0.97 ± 0.02) values (Table 2).

### Integration of significant metabolites and protein mediators

**Fig 3:**
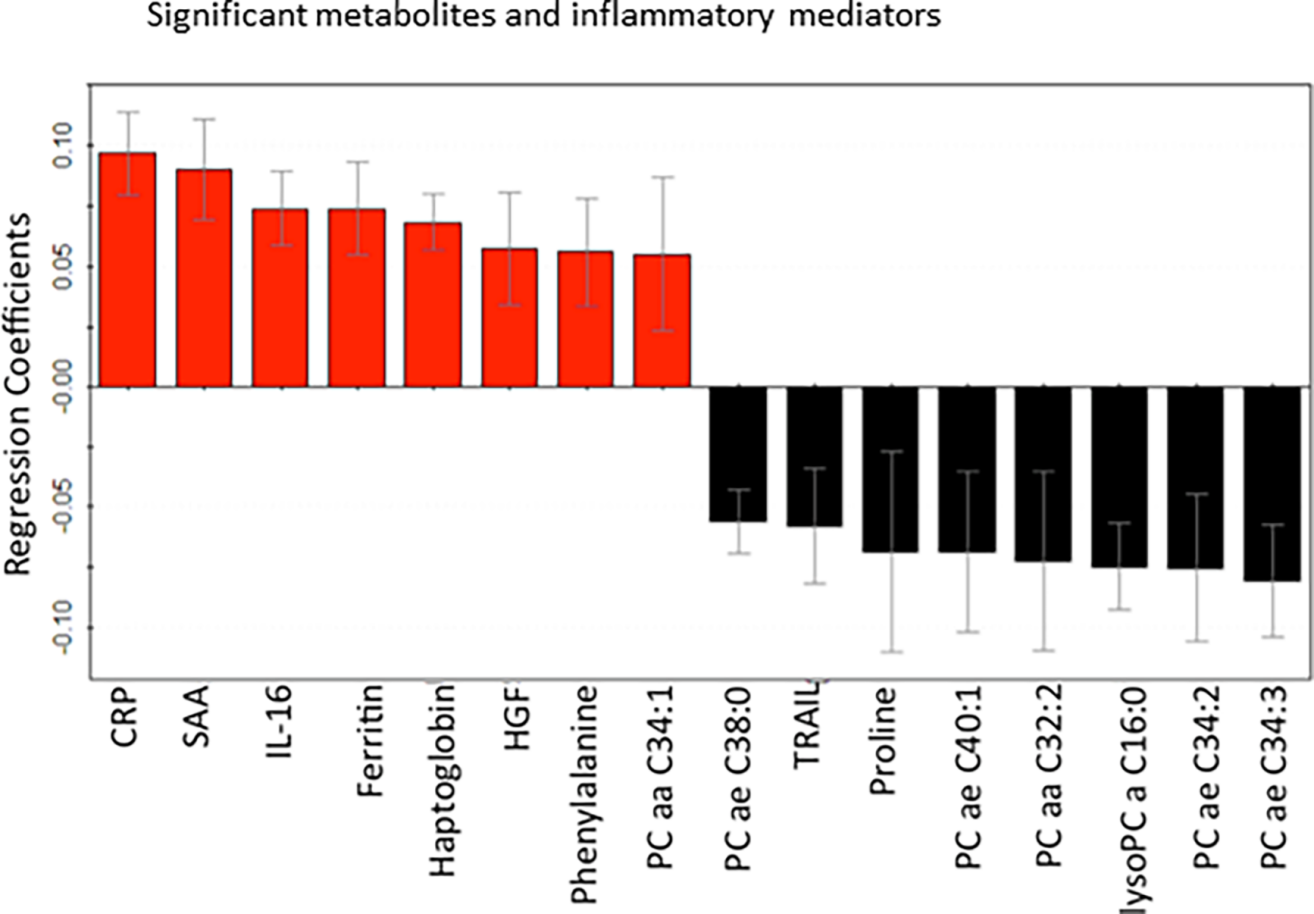
The regression coefficient plots for the statistically significant (p <0.05) metabolites and inflammatory mediators distinguishing children with and without appendicitis. Red bars with positive coefficient values represent increased concentrations in the appendicitis samples, and black bars with negative values represent reduced concentration in the appendicitis patients, compared with control samples. Abbreviations: PC-Phosphatidylcholine, SAA-Serum amyloid A, CRP-C-reactive protein, HGF-Hepatocyte growth factor, IL-16-Interleukin-16, and TRAIL-Tumor necrosis factor-related apoptosis-inducing ligand.

A total of 9 metabolites and 7 cytokine/chemokine compounds (Table 3) provided the most significant contribution for the separation between appendicitis and control patient groups in the corresponding OPLS-DA models. The metabolites comprised of the amino acids phenylalanine and proline, and seven phosphocholines. The inflammatory compounds included serum amyloid A (SAA), C-reactive protein (CRP), ferritin, haptoglobin, hepatocyte growth factor (HGF), interleukin-16 (IL-16), tumor necrosis factor TNF-related apoptosis-inducing ligand (TRAIL) (Fig 3). These 16 compounds were combined to develop an integrated metabolic and cytokine/chemokine profile. The unsupervised PCA-X model was built on a single component PC1 (Fig 1C). One outlier belonging to the appendicitis patient group was excluded from the supervised OPLS-DA model, which separated the two groups with strong variance (cumulative R^2^Y = 0.69) and predictivity (cumulative Q^2^ = 0.67) (Fig 2C). The values calculated for sensitivity (81%), specificity (100%), and AUROC (0.96 ± 0.03) were also substantially high (Table 2).

**Table 3:** List of the most significant metabolites and inflammatory protein mediators identified in children with appendicitis.

Metabolites	Inflammatory protein mediators
Phosphatidylcholine (6 types)	C-reactive protein (CRP)
Lysophosphatidylcholine	Serum amyloid A (SAA)
Phenylalanine	Interleukin-6 (IL-6)
Proline	Ferritin
	Haptoglobin
	Hepatocyte growth factor (HGF)
	Tumor necrosis factor-related apoptosis-inducing ligand (TRAIL)

As previously mentioned only two, three and one outliers were identified in the PCA analyses; to ensure that the exclusion of these outliers from the supervised analyses did not affect the results, we re-developed the OPLS-DA models including all outliers, for all the datasets. Comparable results from the re-analysis demonstrated that the exclusion did not bias the findings (Table 4).

**Table 4:** Comparison of R^2^Y and Q^2^ values calculated for the OPLS-DA models with and without excluding the outliers.

Dataset	R^2^Y Q^2^ Outliers included	R^2^Y Q^2^ Outliers excluded
Metabolomics	0.75 0.54	0.74 0.54
Inflammatory mediator	0.77 0.70	0.75 0.63
Combined profile	0.71 0.69	0.69 0.67

## Discussion

Our pilot study demonstrated an initial step of applying integrated metabolomics and inflammatory biomarker profiling as a potential tool to improve the diagnosis of appendicitis. Our metabolomics analysis using ^1^H-NMR spectroscopy and FIA MS/MS techniques evidently differentiated pediatric patients with appendicitis from pediatric control patients, which is supported by the very goof variance and predictive values (R^2^Y = 0.74 and Q^2^ = 0.54) in the supervised model. Similar notable separation between diseased and control patient groups was observed from the Luminex analysis of the cytokine mediators (R^2^Y = 0.75 and Q^2^ = 0.63) as well. Integration of the significant metabolites and cytokine/chemokine compounds stabilized the supervised model, which is demonstrated by the lowered difference between the variance and predictive values (R^2^Y = 0.69 and Q^2^ = 0.67) compared to the individual profiles (Table 2). Our findings also illustrated sensitivity, specificity and AUROC values from the metabolic and protein mediator profiles, both separate and combined, were high enough to suggest that the approach could distinguish children with appendicitis from those without. Future studies comparing larger cohorts of children with abdominal pain caused by appendicitis to those with abdominal pain not caused by appendicitis will strengthen the validity of our results.

The 9 metabolites and 7 inflammatory mediators detected to be important in distinguishing appendicitis from non-appendicitis suggest that appendicitis might stimulate an expansive alteration in the metabolic and immune processes of the children. MetaboAnalyst 3.0 was used to identify the metabolic pathways perturbed during appendicitis [38]. About 43% of the significant compounds comprised of phosphatidylcholines (Fig 3), a group of phospholipids with choline headgroup, indicating major disruptions in the glycerophospholipid and choline metabolism pathways [38–40]; the presence of phenylalanine, and proline in the metabolite list implies that appendicitis also alters metabolism of amino acids phenylalanine, tyrosine, tryptophan, arginine, and proline [38–40]. In addition, identification of several protein mediators infers the alteration of inflammatory processes by appendicitis [39,40]. Elevated levels of the acute phase proteins serum amyloid A (SAA) and C-reactive protein (CRP) in appendicitis patients are consistent with previous studies; CRP is already an established biomarker that is often used in laboratory testing and clinical scoring of appendicitis [41–46] and SAA is a strongly suggested one [47–49]. In agreement with our findings, the gene encoding the mediator haptoglobin was found overexpressed in the serum of appendicitis patients [48]. Overall, the metabolites and inflammatory mediators detected in this study provide an explicit biomarker pattern that might be used to better diagnose appendicitis among pediatric patients and better understand the underlying pathophysiology.

The results of our study have important future diagnostic implications in clinical settings where children present with abdominal pain. Time is a very crucial factor for appendicitis diagnosis, as a delay can lead to perforation, which can in turn cause severe complications. However, the delay is often unavoidable in children due to the lack of conventional signs and symptoms, and the aforementioned barriers in taking clinical history [1,2,14,15]. Biomarker phenotyping is a relatively fast approach that can potentially address the issue of delayed diagnosis; the presence of small number of metabolites and inflammatory biomarkers in the identified pattern makes it possible to develop a time-sensitive diagnostic aid for the emergency healthcare professionals. Similar metabolomic and inflammatory biomarker phenoyping approach has been successfully applied to early diagnosis and prognosis of a more time-sensitive and fatal pediatric emergency, sepsis, the leading cause of mortality among children around the world [26,50].

### Limitations

There were variations in the volumes of some serum samples, which caused slight inconsistencies in the number of control and diseased populations that could be analyzed by the different techniques. For example, a total of 22 appendicitis and 35 control patients were included in metabolic analysis, whereas 23 appendicitis and 34 control patients were included in protein mediator analysis. Hence, during integration of the profiles we could include only the patients common in both profiles (22 appendicitis and 34 control). However, we believe, the slight inconsistency should not significantly affect our findings. As mentioned previously, this initial pilot study compares children with a diagnosis of appendicitis to controls without abdominal pain; our intent is to provide preliminary data demonstrating a specific metabolomic and inflammatory mediator signal found in children with appendicitis. The validity of our approach will require testing in a the real-world clinical setting by evaluating children presenting to the ED with undifferentiated abdominal pain and determining if the biomarker profiling separates those with and without pathology proven appendicitis. The results from our pilot study are encouraging and demonstrate the feasibility of more robust, larger-scale investigations. Finally, current metabolomics and Luminex technologies are often not available in the ED setting; future industrial partnerships to develop rapid biomarker phenotyping assays will improve integration of our results into the ED workflow.

## Conclusions

Children with appendicitis appear to have a distinct metabolomic and inflammatory mediator profile. This biomarker pattern, comprised of a small set of metabolites and inflammatory mediators, provides insight into the underlying pathophysiologic processes of disease and has the potential to be used for the development of a clinical tool for aiding the diagnosis of pediatric appendicitis. Further multi-centre studies on larger and independent patient cohorts will be needed to validate our findings. Other future directions could include biomarker phenotyping for the stratification of children with appendicitis based on severity of disease (simple versus perforated).

## Supporting information

S1 Table List of the detected metabolites (n = 144) and inflammatory protein mediators (n = 56). (XLSX)
